# Recurrent Vulvovaginal Candidosis and Cluster Analysis of Clinical Signs and Symptoms: A Laboratory-Based Investigation

**DOI:** 10.3390/jof6030113

**Published:** 2020-07-22

**Authors:** Aleksandra Ignjatović, Valentina Arsić-Arsenijević, Milan Golubović, Saša Đenić, Stefan Momčilović, Aleksandra Trajković, Marina Ranđelović, Vojislav Ćirić, Suzana Otašević

**Affiliations:** 1 Department of Medical Statistics and Informatics, Faculty of Medicine, University of Niš, 18000 Niš, Serbia; aleksandra.ignjatovic@medfak.ni.ac.rs; 2 Public Health Institute Niš, 18000 Niš, Serbia; marina87nis@gmail.com (M.R.); otasevicsuzana@gmail.com (S.O.); 3 Department of Microbiology and Immunology, Faculty of Medicine, University of Belgrade, 11000 Belgrade, Serbia; 4 Faculty of Medicine, University of Niš, 18000 Niš, Serbia; milanpfc@gmail.com (M.G.); saska0906@gmail.com (S.Đ.); m-stefan@mts.rs (S.M.); 5 Clinic of Pediatrics, Clinical Center Niš, 18000 Niš, Serbia; 6 Center for Radiology, Clinical Center Nis, 18000 Niš, Serbia; 7 Plastic and Reconstructive Surgery Clinic, Clinical Center Niš, 18000 Niš, Serbia; 8 Faculty of Medicine, University of Belgrade, 11000 Belgrade, Serbia; alekstrajkovic10@gmail.com; 9 Department of Microbiology and Immunology, Faculty of Medicine, University of Niš, 18000 Niš, Serbia; 10 Department of Internal Medicine, Faculty of Medicine, University of Niš, 18000 Niš, Serbia; ciricv@yahoo.com

**Keywords:** *Candida*, recurrent vulvovaginal candidiasis, quality of life, signs and symptoms, cluster analysis

## Abstract

Recurrent vulvovaginal candidosis (RVVC) represents a major health problem that significantly affects a patient’s quality of life (QoL). This infection presents with a plethora of clinical manifestation, and this is the first study that carries out a cluster analysis of these signs and symptoms (SS). The goals are to evaluate the distribution of species causing RVVC, their in-vitro susceptibility to antifungals, and the patient’s QoL. Additionally, the clinical characteristics are analyzed using cluster analysis. Prospective analysis of data was performed for women diagnosed with RVVC in the period from January 2016 to December 2019 based on the analysis of data from a single-center’s records. The standard mycological methods and antifungal susceptibility testing were done. Clinical characteristics and QoL were examined by appropriate questions. The cluster analysis was used to identify clusters of SS. A total of 320 women were diagnosed. The dominant species was *Candida (C.) albicans*. Non-*albicans Candida* (NAC) yeast was found in 24.4%, and the most common was *C. glabrata*. Interestingly, *Saccharomyces (S.) cerevisiae* was detected in 2%. All of the isolated species, except *C. parapsilosis* and *C. kefyr,* demonstrated reduced susceptibility to antifungals. We confirmed the emergence of the NAC species and *S. cerevisiae* with reduced susceptibility to antifungals. Cluster analysis represented by a dendrogram revealed three SS clusters: irritation, uncommon, and discharge, but further studies are needed to examine the relationship between clusters, *Candida* strains, and outcomes.

## 1. Introduction

Vulvovaginal candidosis (VVC) is one of the most common infections in women, with prevalence between 15% and 30% [1], though even higher prevalence rates were noted in some countries [2]. Furthermore, it is assumed that 70–75% of women suffer at least one episode of VVC in their lifetime [1]. All this is understandable since molecular studies have proven that vulvovaginal *Candida*-colonization is present in up to 64% of women [3,4].

Another problem is the fact that about 9% of the female population suffer from a recurrent form of VVC [5], which is defined as more than three symptomatic *Candida* genital infections over a one-year period [1]. The recurrent vulvovaginal candidosis (RVVC) requires more attention from the gynecologists, with an individual approach to each woman. Moreover, it is desirable to perform mycological analyses with antifungal susceptibility testing for every case [6,7]. In addition, contributing factors, namely, intestinal *Candida*-overgrowth and sexual transmission, should also be excluded.

Clinically, VVC can be manifested as a nonspecific genital infection with a plethora of signs and symptoms (SS) such as discharge, itching, burning, and erythema. However, it can also be a complicated condition, such as RVVC, characterized by chronicity or pronounced SS [8].

It is generally known that predominant species causing this infection is *C. albicans* [6]. However, in recent years, non-*albicans Candida* (NAC) species, such as *C. glabrata* or *C. krusei,* have been increasingly recognized as the cause of both sporadic and recurrent VVC [9,10]. Moreover, in the literature, there is evidence that yeast *S.cerevesiae* could be the causative agent of genital fungal infection as well [11,12].

Having in mind the impact of RVVC on global health, the increasing prevalence of this infection in women, as well as the emergence of *Candida*-resistance to commonly used antifungal drugs, we analyzed women with RVVC in order to determine the most frequent causative agents of RVVC and in-vitro susceptibility to antifungals. Additionally, using cluster analysis, SS of women with proven RVVC were evaluated for the first time.

## 2. Materials and Methods

The research was designed as a single-center prospective study. In the period from 2016 to 2019, women with four or more clinically and laboratory-confirmed episodes of fungal genital infection in the previous year were included in the study in their relapse period. Vaginal swabs were analyzed both bacteriologically and mycologically. In addition, a parasitological examination was performed. 

The isolation and identification of yeast were done using standard mycological procedures. Fungal growth material was inoculated in Sabouraud dextrose agar (SDA; Liofilchem Diagnostici, Roseto degli Abruzzi, Italy) and chromogenic *Candida* media (Liofichem/Bacteriology products, Roseto degli Abruzzi, Italy) (both incubated at 37 °C for up to 7 days). Species of genus *Candida* were identified using the germ tube test, the chromogenic *Candida* media (Liofichem/Bacteriology products, Roseto degli Abruzzi, Italy), and Auxacolor^TM^ (BioRad, Marnes-la-Coquette, France). 

A commercial assay for in vitro antifungal susceptibility testing—Fungifast (ELITech Microbiology Reagents, Puteaux, France) and Fungitest^TM^ (BioRad, Marnes-la-Coquette, France)—was used to determine the efficiency of amphotericin B (AmB) in a concentration of 0.5, 2, and 8 µg/mL; 5-fluorocytosine (5-FC) in a concentration of 2, 4, 16, and 32 µg/mL; itraconazole (ITZ) in a concentration of 0.125, 0.5, and 4 µg/mL, fluconazole (FCZ) in a concentration of 8, 32, and 64 µg/mL; voriconazole (VRZ) in a concentration of 1 µg/mL; ketoconazole (KCZ) in concentration of 0.5 and 4 µg/mL; and miconazole (MCZ) in concentration of 0.5 and 8 µg/mL. The effectiveness of tested antimicrobials is interpreted on the basis of their determined minimal inhibitory concentrations (MIC) and the manufacturer’s recommendations, which based on EUCAST discussion document E. Dis. 7.1.: “Method for determination of MIC by broth dilution of fermentative yeasts” established by Subcommittee of Antifungal Testing of the European Committee on Antibiotic Susceptibility of European Society of Clinical Microbiology and Infectious Diseases 2002. 

The Ethical Committee of the University of Niš, Faculty of Medicine (decision No. 12-6316-2/1-2016, 16/06/2016), approved this research. It was conducted according to the ethical guidelines of the Declaration of Helsinki and the ethical policies of the journal, as noted on the journal’s author guidelines page. Written informed consent was obtained from all study participants.

### 2.1. Clinical Characteristics and Quality of Life (QoL) Assessment 

Data regarding clinical characteristics were collected by a gynecologist who filled in a semistructured questionnaire. All women with a laboratory-based diagnosis of RVVC and with SS of infection during the physician’s examination were included in the study. The study of the clinical characteristics and QoL also involved 109 control subjects (mean age 30.73 ± 9.3) who were healthy women who came for a control examination without a history of RVVC, pregnancy, or use of oral contraceptives or corticosteroids.

The Serbian version of self-completion generic questionnaires EuroQol-5 Dimension (EQ-5D) and EuroQol-VAS (EQ-VAS), developed by the EuroQoL Group [13], was used for the assessment of a patient’s QoL. The modified EQ-5D was used for the assessment of usual activities, pain/discomfort, and anxiety/depression, while instead of mobility and self-care, sexual problems and self-confidence were evaluated. Respondents described these five features as having no problems, some problems, or severe problems. Women rated their health status using EQ-VAS, a vertical, visual analog scale, where 0 points represent "the worst" and 100 points "the best imaginable” health states. This instrument provided us with a quantitative measure of the patients’ wellbeing. 

### 2.2. Statistical Analysis 

Continuous data are presented as arithmetic mean ± standard deviation (SD), and categorical variables are expressed as number (n) with percentage (%). The *t*-test was used for comparison of the continuous variables between the groups. Intergroup comparisons of categorical variables were made using the chi-square test. The agglomerative hierarchical cluster analysis with Ward’s method was used to identify SS clusters, with the Euclidean distance being used to measure the similarity between variables. Clustering results are displayed with the dendrogram. Women suffering from RVVC were allocated to one of the three groups (having all of the SS, having some of the SS, or having none of the SS of that cluster) for each of the established SS clusters. All statistical analyses were performed using R software (version 3.4.3; The R Foundation for Statistical Computing, Austria) [14]. The significance level was set to *p* < 0.05.

## 3. Results

### Patients Characteristics and Laboratory Analysis

In total, 320 women were diagnosed with RVVC during the study period (mean age 32.03 ± 5.6). The most common causative agent of RVVC was *C. albicans* (73.8%). In the group of NAC species, the most prevalent were *C. glabrata* (13.8%) and *C. krusei* (6.3%). On the other hand, species *C. tropicalis* (2.2%), *C. parapsilosis* (1.3%), and *C. kefyr* (0.9%) were proven in samples of considerably fewer women. *S. cerevesiae* as causative agent of RVVC was found in six patients (1.9%; Table 1).

Evaluation of antifungal susceptibility obtained by the two commercial tests showed that a high percentage of *C. albicans* isolates were sensitive to AmB (95.3%), 5-FC (89.4%), and KTZ (90.3%). In contrast, the higher MIC^ITZ^ = 0.25–0.5 µg/mL, MIC^FCZ^ = 16–32 µg/mL, MIC^VRZ^ >1 µg/mL, and MIC^MCZ^ >0.5 µg/mL was established in 65.7%, 33.5%, 22.5%, and 46.2%, respectively. Similarly, *C. glabrata* isolates had a lower sensitivity to triazoles, especially to itraconazole and fluconazole, since 75.0% of this NAC species were intermediately sensitive to these drugs. Additionally, MCZ showed lower efficiency, wherein 72.7% MIC^MCZ^ was higher than 0.5 µg/mL. As for *C. krusei*, which was expected, all isolates were resistant to FCZ, followed by a very high percentage of this strain with low sensitivity to ITZ (75.0%) and MCZ (40.0%). *C. tropicalis* isolates in a high percentage had low sensitivity to ITZ (57.1%), FCZ (71.4%), and MCZ (71.4%). Two isolates of *S. cerevesiae* (33.3%) had lower susceptibility to all included antifungals except 5-FC. In contrast, all isolates of *C. parapsilosis* and *C. kefyr* showed satisfactory sensitivity to applied antifungals (Table 2).

All of the investigated SS, except unpleasant smells, were significantly more common in women with RVVC (Table 3).

Most frequently, these women complained about increased and whitish discharge, which disturbed 78.8% and 71.2% of infected women, respectively. On the other hand, edema was the least common SS, noted in only 10% of patients. 

**Figure 1:**
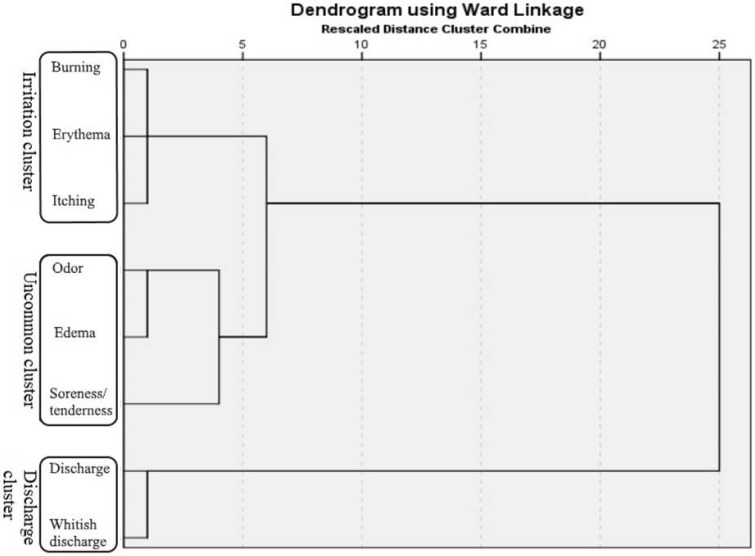
Dendrogram of the recurrent vulvovaginal candidiasis (RVVC) signs and symptoms clusters (Ward method; binary measure—Euclidian distance).

Signs and symptoms were then subjected to cluster analysis. The three-cluster solution was elected as optimal, based on the dendrogram (Figure 1). Signs and symptoms commonly associated with vaginal infection, burning, erythema, and itching, were clustered together into a cluster we named the "irritation cluster". The second cluster also comprised three SS: unpleasant smell, edema, and soreness/tenderness. This cluster of atypically grouped SS was named the "uncommon cluster". The third cluster consisted of two SS: excessive and whitish discharge, so it was labeled as the "discharge cluster". Forty-eight women presented with all SS of the "irritation cluster", while only 4 women presented with all SS of the "uncommon cluster". Both SS of the "discharge cluster" occurred in 208 women; therefore, this was the most common cluster (Table 4). 

There were no statistical differences in the frequency of presence of SS in any of the three clusters (*p* = 0.382, *p* = 0.249, *p* = 0.102) when comparing *C. albicans* and the NAC species (included *S. cerevisiae*). 

The assessment of the QoL data showed that usual activities, pain/discomfort, self-confidence, and sexual problems were equally present in the examined groups. The statistically higher number of subjects without anxiety/depression was the RVVC group compared to the control group (38.8% vs. 16.5%, *p* = 0.001). Values of the EQ-VAS score were not significantly different between RVVC and the control group (73.9 ± 15.2 vs. 74.2 ± 14.7, *p* = 0.862)

## 4. Discussion

The prevalence of RVVC of women has increased over the past years. Regardless of numerous treatments that seem beneficial and effective, a large portion of women develop a recurrence of the infection that can have an impact on the patient’s QoL and self-confidence. One of the concepts emphasizes the role of the decreased sensitivity of yeast to applied antifungal therapy conjoined with reduced vaginal defense mechanisms [15].

Species *C. albicans* still remains the dominant species identified in over 70% of cases. However, confirmed NAC-RVVC shows remarkably higher prevalence in comparison to the last 20 years when they were found in less than 5% of women [16]. The most commonly isolated species were *C. glabrata* (13.8%) and *C. krusei* (6.3%). Rarely, etiological agents of RVVC were *C. tropicalis* (2.2%), *C. kefyr* (0.9%), and *C. parapsilosis* (1.3%). It is assumed that low-dose systemic antifungal therapy, single-dose local antifungal therapy, as well as possibility of self-treatment easily available to everyone have contributed not only to high prevalence of NAC-RVVC but also to their decreased susceptibility to antifungals [1,12].

In addition to the rising prevalence of non-*Candida* RVVC, a "new" cause of fungal genital infection is emerging—species *S. cerevisiae.* This yeast is a part of the physiological microbiota of respiratory, intestinal, and vaginal mucosa and was, until recently, considered nonpathogenic. Although genital infections caused by *S. cerevisiae* are still only occasionally reported, in our study, this species was responsible for about 2% of RVVC in women. Following the SS of *S. cerevisiae* infection, it was determined that all patients have increased and milky-white secretion as the dominant clinical finding, which is no different from a *Candida* infection. Research performed in Greece has even found that this fungi is the third most common cause of RVVC [11]. Similar to NAC-RVVC, *S. cerevisiae* infection is also associated with a higher risk of recurrence and shows decreased sensitivity to antifungals [17,18].

According to the fact that low sensitivity to applied therapy could be the cause of relapse, in our study, all isolates were tested by two commercial antifungal susceptibility tests. These kits for antifungal susceptibility testing can be used as a screening test for the detection of antifungal efficacy. Results of in vitro testing showed that in a very high percentage of women where *C. albicans* or *C. glabrata* were the causative agents, triazoles and MCZ did not have satisfactory effectiveness since the determined MICs for this drugs were higher. The mentioned finding was followed by lower sensitivity to antifungals of *C. krusei* and *C. tropicalis*. Species *C. krusei* is already a recognizable resistant-species to FCZ, and in this study, besides this, it showed lower sensitivity to ITZ in a high percentage as well. Similarly, a big portion of *C. tropicalis* isolates demonstrated low susceptibility to triazoles and MCZ. Only two species, *C. parapsilosis* and *C. kefyr,* were sensitive to all applied antifungal drugs, but these isolates were established in low percentage of women. As for the "new" causative agent of fungal genital infection, *S. cerevisiae,* it can be pointed out that it could be a problem in treatment since the two isolates showed low sensitivity to all included antifungals except 5-FC. 

Contrary to our results, research that included over 3000 strains of *Candida* spp. isolated from women suffering from VVC did not note the high prevalence of samples with decreased susceptibility to antifungals [19]. This discrepancy can be explained by the fact that the mentioned research did not include women with RVVC. Such an assumption is supported by studies that compared the sensitivity of strains detected in RVVC and VVC women. These studies found that in-vitro tested antifungals had lower efficiency on the isolates from RVVC women [12,20,21,22].

When it comes to SS of RVVC, it is shown that they are the same as the ones that occur in acute VVC [23], including itching, discharge, burning, and erythema [8,24]. These SS occur with different prevalence in different studies [23,25].

Patients with RVVC experienced almost all of the examined SS significantly more often compared to the control. Only the presence of odor did not differ significantly between groups. Given the fact that it is a highly subjective sign, this should not surprise. The dominant SS among our patients was an increased and whitish discharge, which was also observed by research done in Italy [25].

Since RVVC rarely presents with a single, but rather, with numerous SS simultaneously, SS cluster analysis was performed. This meant the creation of SS cluster from scratch, with no clue of what to expect, since this is the first such analysis on the subject. Three clusters were formed and labeled according to the SS they comprised. Thus, we had irritation, uncommon, and discharge clusters. Irritation and discharge clusters both included SS that usually appear together and are considered typical for infection. The uncommon cluster, as its name says, included SS that rarely appear together, suggesting that even though it was the rarest of the three, it can occur and mislead clinicians. For this reason, skipping steps in the diagnostic algorithm of RVVC and leaning only on the SS is not advisable. This shortcut, alongside self-diagnosed RVVC, carries the risk of the wrong treatment or overtreatment of the patient’s condition. Additionally, the analysis of the presence of different species as causative agents of RVVC did not show a statistically significant difference in examined clusters.

The quality of life survey revealed that the EQ-VAS score is not significantly different among groups. Even though the control group had a slightly better EQ-VAS score than RVVC, no significant difference was presented, suggesting that RVVC does not crucially influence daily routine. 

The limitation of the study is the usage of screening tests for the determination of antifungal susceptibility in the absence of an established standardized dilution test. Additionally, the use of more specific questionnaires might provide better insight into the patient’s QoL. This study shows the emergence of RVVC caused by non-*Candida* species such as *S. cerevisiae*. Therefore, new definitions and new therapeutic strategies are required, mainly because these species show reduced sensitivity to antifungals. As RVVC is still a complex medical topic, the performance of the cluster analysis may be useful for application to laboratory characteristics, separately or in connection with clinical data; further studies are needed.

## Figures and Tables

**Table 1:** Prevalence of recurrent vulvovaginal candidosis (RVVC) caused by *Candida* spp. and *S. cerevesiae.*

Causative Agent of RVVC	n (%)
*C. albicans*	236 (73.8)
*C. glabrata*	44 (13.8)
*C. krusei*	20 (6.3)
*C. tropicalis*	7 (2.2)
*C. parapsilosis*	4 (1.3)
*C. kefyr*	3 (0.9)
*S. cerevesiae*	6 (1.9)

Abbreviations: C—*Candida*; S—*Saccharomyces*.

**Table 2:** Antifungal susceptibility testing of fungi caused RVVC obtained by Fungifast^TM^ and Fungitest^TM.^

Species	Antifungal Susceptibility Testing, n (%)															
	5-FC 2–32 µg/mL		AmB 0.5–8 µg/mL			ITZ 0.125–4 µg/mL		FCZ 8–64 µg/mL			VRZ 1 µg/mL		KCZ 0.5–4 µg/mL		MCZ 0.5–8 µg/mL	
	MIC ≤ 4 µg/mL	MIC = 8–16 µg/mL	MIC ≤ 0.5 µg/mL	MIC = 2 µg/mL	MIC > 2 µg/mL	MIC ≤ 0.125 µg/mL	MIC = 0.25–0.5 µg/mL	MIC ≤ 8 µg/mL	MIC = 16–32 µg/Ml	MIC > 32 µg/mL	MIC ≤ 1 µg/mL	MIC > 1 µg/mL	MIC < 0.5 µg/mL	MIC > 0.5 µg/mL	MIC < 0.5 µg/mL	MIC > 0.5 µg/mL
	S	I/ SDD	S	I/ SDD	R	S	I/ SDD	S	I/ SDD	R	S	I/ SDD	S	I/ SDD	S	I/ SDD
*C. albicans*	211 (89.4)	25 (10.6)	225 (95.3)	11 (4.7)	0	81 (34.3)	155 (65.7)	157 (66.5)	79 (33.5)	0	183 (77.5)	53 (22.5)	213 (90.3)	23 (9.7)	127 (53.8)	109 (46.2)
*C. glabrata*	43 (97.7)	1 (2.3)	44 (100)	0	0	11 (25)	33 (75)	10 (22.7)	33 (75)	1 (2.3)	31 (70.5)	13 (29.5)	39 (88.6)	5 (11.4)	12 (27.3)	32 (72.7)
*C. krusei*	19 (95)	1 (5)	18 (90)	0	2 (10)	5 (25)	15 (75)	0	0	20 (100)	19 (95)	1 (5)	19 (95)	1 (5)	12 (60)	8 (40)
*C. tropicalis*	7 (100)	0	7 (100)	0	0	3 (42.9)	4 (57.1)	2 (28.6)	5 (71.4)	0	7(100)	0	7 (100)	0	2 (28.6)	5 (71.4)
*C. parapsilosis*	4 (100)	0	4 (100)	0	0	4 (100)	0	4 (100)	0	0	4 (100)	0	4 (100)	0	4 (100)	0
*C. kefyr*	3 (100)	0	3 (100)	0	0	3 (100)	0	3 (100)	0	0	3 (100)	0	4 (100)	0	4 (100)	0
*S. cerevesiae*	6 (100)	0	4 (66.7)	0	2 (33.3)	4 (66.7)	2 (33.3)	4 (66.7)	2 (33.3)	0	4 (66.7)	2 (33.3)	4 (66.7)	2 (33.3)	4 (66.7)	2 (33.3)

Abbreviations: RVVC—recurrent vulvovaginal candidiasis; C—*Candida*; S—*Saccharomyces*; MIC—minimal inhibitory concentrations; S—sensitive; I—intermediate; SDD—susceptible dose-dependent; R—resistant; AmB—amphotericin B; 5-FC—5-fluorocytosine; ITZ—itraconazole; FCZ—fluconazole; VRZ—voriconazole; KCZ—ketoconazole; MCZ—miconazole.

**Table 3:** Clinical data between the examined groups.

Characteristics	RVVC, n (%)		Control, n (%)		*p* ^*^
**Itching**					
**0**	216	(67.5)	98	(89.9)	<0.001
**1**	104	(32.5)	11	(10.1)	
**Discharge**					
**0**	68	(21.2)	61	(56.0)	<0.001
**1**	252	(78.8)	48	(44.0)	
**Odor**					
**0**	284	(88.8)	100	(91.7)	0.484
**1**	36	(11.2)	9	(8.3)	
**Burning**					
**0**	232	(72.5)	104	(95.4)	<0.001
**1**	88	(27.5)	5	(4.6)	
**Erythema**					
**0**	260	(81.2)	104	(95.4)	<0.001
**1**	60	(18.8)	5	(4.6)	
**Whitish discharge**					
**0**	92	(28.8)	63	(57.8)	<0.001
**1**	228	(71.2)	46	(42.2)	
**Edema**					
**0**	288	(90.0)	108	(99.1)	0.004
**1**	32	(10.0)	1	(0.9)	
**Soreness/tenderness**					
**0**	224	(70.0)	99	(90.8)	<0.001
**1**	96	(30.0)	10	(9.2)	

Abbreviations: RVVC—recurrent vulvovaginal candidiasis. * Chi-square test; 0—without symptom/sign; 1—experiencing.

**Table 4:** Presence of signs and symptoms (SS) among clusters of patients

Signs and Symptoms	Irritation Cluster, n (%)		Uncommon Cluster, n (%)		Discharge Cluster, n (%)	
None	188	(58.8)	200	(62.5)	48	(15)
Some	84	(25.5)	116	(36.3)	64	(20)
All	48	(15)	4	(1.3)	208	(65)
	320	(100)	320	(100)	320	(100)

## References

[1] Sobel J.D. (2007). Vulvovaginal candidosis. Lancet.

[2] Bitew A., Abebaw Y. (2018). Vulvovaginal candidiasis: Species distribution of Candida and their antifungal susceptibility pattern. BMC Womens Health.

[3] Cauchie M., Desmet S., Lagrou K. (2017). Candida and its dual lifestyle as a commensal and a pathogen. Res. Microbiol..

[4] Drell T., Lillsaar T., Tummeleht L., Simm J., Aaspõllu A., Väin E., Saarma I., Salumets A., Donders G.G.G., Metsis M. (2013). Characterization of the vaginal micro- and mycobiome in asymptomatic reproductive-age Estonian women. PLoS ONE.

[5] Denning D.W., Kneale M., Sobel J.D., Rautemaa-Richardson R. (2018). Global burden of recurrent vulvovaginal candidiasis: A systematic review. Lancet Infect. Dis..

[6] Tasić S., Miladinović-Tasić N., Tasić A., Zdravković D., Djordjević J. (2008). Exogenic reinfection—A possible cause of recurrent genital candidosis in women. Acta Fac. Med. Naissensis.

[7] Tasić S., Miladinović–Tasić N., Tasić A. (2003). Endogenous reinfection as a cause of recurrent GC in women. Srp. Arh. Celok. Lek..

[8] Blostein F., Levin-Sparenberg E., Wagner J., Foxman B. (2017). Recurrent vulvovaginal candidiasis. Ann. Epidemiol..

[9] Otašević S., Momčilović S., Trajkovic A., Arsic-Arsenijevic V. Modelling of antifungal treatment with azoles and essential oils for non-albicans *Candida* spp. causing vulvo-vaginal infections. Presented at the 27th European Congress of Clinical Microbiology and Infectious Diseases.

[10] Makanjuola O., Bongomin F., Fayemiwo S.A. (2018). An update on the roles of non-albicans Candida species in vulvovaginitis. J. Fungi (Basel).

[11] Papaemmanouil V., Georgogiannis N., Plega M., Lalaki J., Lydakis D., Dimitriou M., Papadimitriou A. (2011). Prevalence and susceptibility of Saccharomyces cerevisiae causing vaginitis in Greek women. Anaerobe.

[12] Mendling W., Brasch J., Cornely O.A., Effendy I., Friese K., Ginter-Hanselmayer G., Hof H., Mayser P., Mylonas I., Ruhnke M. (2015). Guideline: Vulvovaginal candidosis (AWMF 015/072), S2k (excluding chronic mucocutaneous candidosis). Mycoses.

[13] EuroQoL Group (1990). EuroQoL—A new facility for the measurement of health-related quality of life. Health Policy.

[14] R Core Team (2013). R: A Language and Environment for Statistical Computing. R Foundation for Statistical Computing, Vienna, Austria. http://www.R-project.org/.

[15] Tasić S., Miladinović-Tasić N. (2009). Immunopathogenesis of recurrent GC in women. Med. Pregl..

[16] Tasić S. (1998). Recurrent GC of Women—Microbiological and Immunological Aspects. PhD Thesis.

[17] Echeverría-Irigoyen M.J., Eraso E., Cano J., Gomáriz M., Guarro J., Quindós G. (2011). Saccharomyces cerevisiae vaginitis: Microbiology and in vitro antifungal susceptibility. Mycopathologia.

[18] Savini V., Catavitello C., Manna A., Talia M., Febbo F., Balbinot A., D’Antonio F., Di Bonaventura G., Celentano C., Liberati M. (2008). Two cases of vaginitis caused by itraconazole-resistant Saccharomyces cerevisiae and a review of recently published studies. Mycopathologia.

[19] Liu X.P., Fan S.R., Peng Y.T., Zhang H.P. (2014). Species distribution and susceptibility of Candida isolates from patient with vulvovaginal candidiasis in Southern China from 2003 to 2012. J. Mycol. Med..

[20] Adjapong G., Hale M., Garrill A. (2016). A comparative investigation of azole susceptibility in Candida isolates from vulvovaginal candidiasis and recurrent vulvovaginal candidiasis patients in Ghana. Med. Mycol..

[21] Richter S.S., Galask R.P., Messer S.A., Hollis R.J., Diekema D.J., Pfaller M.A. (2005). Antifungal susceptibilities of Candida species causing vulvovaginitis and epidemiology of recurrent cases. J. Clin. Microbiol..

[22] Hadrich I., Ayadi A. (2018). Epidemiology of antifungal susceptibility: Review of literature. J. Mycol. Med..

[23] Yano J., Sobel J.D., Nyirjesy P., Sobel R., Williams V.L., Yu Q., Noverr M.C., Fidel P.L. (2019). Current patient perspectives of vulvovaginal candidiasis: Incidence, symptoms, management and post-treatment outcomes. BMC Womens Health.

[24] Sobel J.D. (2016). Recurrent vulvovaginal candidiasis. Am. J. Obstet. Gynecol..

[25] Corsello S., Spinillo A., Osnengo G., Penna C., Guaschino S., Beltrame A., Blasi N., Festa A. (2003). An epidemiological survey of vulvovaginal candidiasis in Italy. Eur J. Obstet. Gynecol. Reprod. Biol..

